# Stem cell therapy and FUS[1‐359]‐transgenic mice: A recent study highlighting a promising ALS model and a promising therapy

**DOI:** 10.1111/cns.13302

**Published:** 2020-03-17

**Authors:** Natalia Ninkina

**Affiliations:** ^1^ Institute of Physiologically Active Compounds Russian Academy of Sciences Chernogolovka Russia

Amyotrophic lateral sclerosis (ALS), or Lou Gehrig's disease, is a motor neuron disease (MND), that were first described in 1869 by French neurologist Jean‐Martin Charcot.^1^ This fatal neurodegenerative disorder affects motor neurons and other neuronal cells, leading to severe disability and the death of 80% of sufferers within 5 years. The mechanism that gives rise to the death of neurons in ALS remains unclear, but significant progress has been made in identifying genes that contribute to the pathology and over 20 common genetic mutations of genes, which predominantly encode RNA‐binding proteins (RBPs), have been identified in ALS patients with sporadic (>90%) and familial forms of the disease.^2^

The mutations within superoxide‐dismutase‐1 (SOD‐1) and the fused in sarcoma protein (FUS) are the two most common genetic causes that give rise to ALS. Indeed, over 140 distinct mutations in SOD‐1 have been identified to date. As a consequence, a number of mutant rodent strains have been developed to model ALS based on the SOD‐1. The G93A SOD‐1 mutant line has been most widely studied, but, despite the histopathological similarities between the SOD‐1‐G93A line and the human condition, very few treatments have evolved from our improved understanding of the role of SOD‐1 and effective therapy is still lacking. Among approved standard therapies are Riluzole (Sanofi‐Aventis) that targets TTX‐sensitive sodium channels, NMDA and GABA receptors and Edaravone (Mitsubishi Pharma) ameliorates oxidative cellular stress; both drugs can slow down the disease, but the maximum effect appears to be a 10% reduction in progression. It has become clear from these results that targeting neurons alone is not sufficient to arrest the pathology and that the production of potentially damaging molecules by other cell populations in the brain need to be considered. Inflammation and inflammatory mediators, produced by glial cells, have often been cited as a potential target for ALS therapy given the extensive microglial and astrocyte activation observed in the pathology, but the TNF inhibitor thalidomide, nonsteroid antiinflammatory drugs (NSAIDs), the selective COX‐2 inhibitor celecoxib, corticosteroids, cyclophosphamide, cyclosporine, cytochrome C inhibitors, and caspase‐reducing drugs have all failed to induce an improvement of the ALS pathology.^3^ It is unclear, why these therapies have failed, but it may be owing to systemic vs local effects or the inability of current antiinflammatory interventions to maintain the right balance of neuroprotective cytokines/neurokines in the brain. For example, the suppression of IL‐6 production by antiinflammatory therapy would also result in the loss of the neuroprotective IL‐6/sIL‐6R gp130 interactions,^4^ which may highlights the need for more subtle approaches to the manipulation of the inflammatory response.

The article of de Munter and co‐authors “*Neuro‐Cell therapy improves motor outcomes and suppresses inflammation during experimental syndrome of amyotrophic lateral sclerosis in mice*” 5 has shown that their novel approach has beneficial effect in the G93A SOD‐1 mutant and the more recently described FUS[1‐359] ‐transgenic mouse,^6^, ^7^ which confers a number of advantages over “golden standard” SOD‐1‐G93A line. The study employs a new stem cell preparation known as “Neuro‐Cells” (Neuroplast, Netherlands), which are argued to have novel antiinflammatory actions, such as the suppression of microglial activation, as part of their “stem cell” activity. The effects of the stem cell preparation on weight loss, water and food intake, grip strength, wire hanging, and the rotarod test were examined in addition to histological endpoints. For comparison, separate groups of the FUS[1‐359]‐transgenic mutants were treated with Riluzole or celecoxib and multiple endpoints were also examined. The FUS model recapitulates all key features of ALS such as motor neuron degeneration, muscle atrophy, physiological decline, cachexia, and neuroinflammation. Importantly, in this study, these features were, to some extent, sensitive to the standard treatments with Riluzole and celecoxib, which served to validate the claim that the FUS[1‐359]‐mice can be used as clinically relevant model of ALS. A relatively narrow time window of the onset of clinical signs also makes it an attractive additional model for further preclinical studies for ALS.

Perhaps that most significant element of the study of de Munter et al is that a stem cell therapy is able to impact on the progression of a neurodegenerative disease model of ALS. It is a generally held view that clinical outcomes for cell transplantation medicines are likely to be unpredictable, even when the preclinical data appear promising; the average length of time from target discovery to approval of a new drug averages ~14 years, and the failure rate exceeds 95%. Because cell‐based therapeutics are more complicated, the usual argument is that clinical performance will be even less predictable. However, recent advances suggest the biomedicines (stem cell therapy for example) are seeing a revolution in the use of human cells as versatile therapeutic engines, for such conditions which are not otherwise treatable. In their work, the authors have demonstrated that an intracerebroventricular administration of their preparation suppresses the impact of the FUS mutation in these mice and also prevents proinflammatory changes associated with the phenotype. Positive effects were observed in all outcome measures. The reference treatments of Riluzole or the antiinflammatory drug celecoxib also exerted similar, but weaker, effects in comparison with those of the stem cells.

The mechanism of action of the Neuro‐Cells preparation in ALS has not been fully investigated, but the combination was selected over other stem cell preparations because it was hypothesized that a benefit would be accrued from the combination of HSCs and MSCs. It has been previously reported that the combination of HSCs and MSCs has an antiinflammatory action in a rat model of spinal cord injury 8 but, given the difference between and acute injury and ALS model pathology, the effects of the stem cells is still surprising. HSCs functions are modulated by a complex interplay between cell‐intrinsic mechanisms and cell‐extrinsic factors produced by the presence of other cells. The MSCs constitute a substantial proportion of Neuro‐Cells preparation and are known to differentiate into a number of cell types and have been shown to contribute to regenerative processes, but they also to play a critical role in the regulation of HSCs function. MSCs maintain HSCs in an undifferentiated state preventing their differentiation, which results in cell aging and death. Thus, the longer‐lived effects observed after the Neuro‐Cells treatment may result from the interaction of the two cell types.

To conclude, the article has highlighted the potential of cell‐based therapy where small molecule treatments have failed. The results are likely to encourage further exploration of stem cells or the extracellular vesicles they produce, or other macromolecular structures from bacteria or viruses^9^ as unique immunomodulators and neuroprotective agents for as yet unresolved medical problems.
